# Triclosan exposure induced disturbance of gut microbiota and exaggerated experimental colitis in mice

**DOI:** 10.1186/s12876-022-02527-z

**Published:** 2022-11-18

**Authors:** Jing Liu, Yang Tao, Wang Haikun, Yang Lanfang, Lu Jingyi, Yao Ping

**Affiliations:** 1 Chinese Academy of Medical Sciences & Peking Union Medical College, 100730 Beijing, China; 2 Department of Gastroenterology, China-Japan Friendship Hospital, 100029 Beijing, China; 3 Department of Gastroenterology, Xinjiang Medical university Affiliated first Hospital, 830000 Urumqi, Xinjiang China

**Keywords:** Triclosan, Gut microbiota, Ulcerative colitis

## Abstract

**Background:**

Triclosan, an antimicrobial agent in personal care products, could be absorbed into the human body through the digestive tract. This animal experiment aimed to clarify the effects of triclosan exposure on the microbiome and intestinal immune functions in healthy and ulcerative colitis models.

**Methods:**

Balb/c mice were maintained on an AIN-93G diet containing 80ppm triclosan dissolved in polyethylene as vehicle or vehicle alone for 1 week or 4 weeks. In the end, the mice were sacrificed, blood samples and colon tissues were collected for analysis of inflammation, and fecal samples were collected for 16 S rRNA sequencing of gut microbiota. To establish ulcerative colitis mice model, at the beginning of the 4th week, mice maintained on the diet with or without triclosan were treated with 2% Dextran sulfate sodium(DSS) in drinking water for 1 week. Then mice were sacrificed for analysis of colitis and gut microbiota.

**Results:**

Triclosan exposure to common mice enhanced the levels of p-NF-κb and Toll-like receptor 4 (TLR4), and decreased the Occludin in the colon. Triclosan exposure to DSS-induced mice increased the level of inflammatory cytokines, reduced the levels of Occludin, and exacerbated the degree of damage to intestinal mucosa and crypt, infiltration of inflammatory cells and atypia of glandular cells. Low-grade intraepithelial neoplasia appeared. Both in common and DSS-induced mice, triclosan exposure changed the diversity and composition of gut microbiota. Fecal samples showed higher enrichment of sulfate-reducing bacteria and Bacteroides, and less butyrate-producing bacteria.

**Conclusion:**

Triclosan exposure induced disturbance of gut microbiota and exaggerated experimental colitis in mice. And changes in the composition of gut microbiota were characterized by the increase of harmful bacteria, including sulfate-reducing bacteria and Bacteroides, and the reduction of protective probiotics, butyrate-producing bacteria.

**Supplementary Information:**

The online version contains supplementary material available at 10.1186/s12876-022-02527-z.

## Background

Ulcerative colitis (UC) is a chronic non-specific inflammatory colorectal disease with unclear pathogenesis. It causes colorectal cancer and the prevalence is over 0.3% at present [1]. The incidence of UC is associated with interactions among immunological response, environmental factors and gut microbiota dysbiosis [2]. A basal gut inflammation environment is fundamental in the initial stage of UC and overzealous inflammatory responses prompt the progression of UC [3]. Triclosan (TCS), as an antimicrobial agent in personal care products, can be absorbed into human body through digestive tract and skin. It was detected in urine, blood and breast milk in people [4, 5]. Previous studies indicated TCS disturbed the balance of gut microbiota in different animals, such as black minnows and mice, even caused colitis [6]. But specific mechanisms of TCS action were not understood. Besides, other research had reverse conclusions [7, 8]. TCS has raised long-standing public concern due to the controversy. This study aimed to clarify the effects of TCS exposure on the microbiome and intestinal immune functions in healthy and UC model.

## Methods

### Study design

The purpose of this study was to clarify the effects of triclosan exposure on the microbiome and intestinal immune functions in healthy and ulcerative colitis model.

We treated mice with 80ppm of TCS, as previously published [4]. When mice were exposed to 80 ppm TCS via diet, the plasma concentration of TCS was 2,422 ± 345 nM and this concentration was comparable to those reported in plasma of TCS-exposed individuals [4, 9, 10]. Next, we treated common mice with TCS for 1 week and 4weeks respectively to observe effects of TCS over the time. Finally, we established a mouse model of UC induced by DSS to explore effects of TCS on UC. For each experiment, the mice were randomized to receive treatments of TCS or vehicle (n = 12/group). All the mice were exsanguinated and sacrificed by removal of the eyeballs at the end.

### Experiment scheme

#### Animal protocols

The detailed animal protocols of each group are shown in Table 1.

**Table 1:** Animal protocols

Study purpose	Groups (n = 12/group)	Treatment method
**Protocol 1**: Effects of TCS exposure for 1 week on gut microbiota and gut inflammation in mice.	1-week TCS vs. 1-week control	Balb/c mice (aged, 4w) were maintained on a diet with 80ppm TCS (97%, Macklin) dissolved in polyethylene glycol 400 (EMD Millipore) as vehicle (0.5% in diet, v/w) or vehicle alone for 1 week. At the end, the mice were sacrificed, blood samples and colon tissue were collected for analysis of inflammation and fecal samples were collected for 16 S rRNA sequencing.
**Protocol 2**: Effects of TCS exposure for 4 weeks on gut microbiota and gut inflammation in mice.	4-week TCS vs. 4-week control	Balb/c mice (aged, 4w) were maintained on a diet with 80ppm TCS (97%, Macklin) dissolved in polyethylene glycol 400 (EMD Millipore) as vehicle (0.5% in diet, v/w) or vehicle alone for 4 weeks. At the end, the mice were sacrificed, blood samples and colon tissue were collected for analysis of inflammation and fecal samples were collected for 16 S rRNA sequencing.
**Protocol 3**: Effects of TCS exposure on gut microbiota and gut inflammation in UC-model mice	DSS + TCS vs. DSS + control	Balb/c mice (aged, 4w) were maintained on a diet with 80ppm TCS (97%, Macklin) dissolved in polyethylene glycol 400 (EMD Millipore) as vehicle (0.5% in diet, v/w) or vehicle alone for 3 weeks. At the beginning of the 4^th^ week, all the mice were treated with 2% DSS in drinking water for 1 week to induce UC, during which the TCS or vehicle treatment remained the same. At the end of the 4^th^ week, the mice were sacrificed, blood samples and colon tissue were collected for analysis of inflammation and fecal samples were collected for 16 S rRNA sequencing.

#### 16 S rRNA sequencing of fecal microbiota

Genomic DNA was extracted from fecal samples using DNA Extraction Kit (DNeasy PowerSoil Kit (100) (QIAGEN,12888-100), QIAamp 96 PowerFecal QIAcube HT kit(5) (QIAGEN,51,531 ), Qubit dsDNA Assay Kit (Life Technologies, Q328520), Takara Ex Taq (Takara, RR001Q)) following vendor’s instructions. NanoDrop and agarose gel electrophoresis were used to determine concentration and purity of gDNA, respectively. Extracted DNA was diluted to a concentration of 1 ng/µl and stored at -20℃ until further processing. The V3-V4 variable region of the 16 S gene was amplified using barcoded PCR with universal primers 343 F and 798 R. Using the V3-V4 front-end primer: 343 F − 5’-TACGGRAGGCAGCAG-3’; and back-end primer: 798R − 5’-AGGGTATCTAATCCT-3’, the PCR was performed using Takara Ex Taq high-fidelity enzyme (Takara Corporation). PCR products were detected by electrophoresis and then purified with AMPure XP beads (Agencourt). The purified products were used in a second PCR round, and again detected by electrophoresis and purified by magnetic beads. Secondary PCR products were quantified with Qubit. Samples were further processed and sequenced at Oebiotech company. Firstly, add the reaction system to the PCR tubes (15 µl 2×Gflex PCR Buffer, 0.6 µl Tks Gflex DNA Polymerase (1.25U/µl), 1 µl Adapter I5, 1 µl Adapter I7, >=1 µl (50ng) First PCR product,

30 µl H2O, 30 µl Total). Then, set up the PCR instrument according to the following procedure (Temperature 94 °C 5 min, 94 °C 30s, 56 °C 30s, 72 °C 20s, 72 °C 5 min, 4 °C hold, 7 cycles). Sequencing analysis was performed by Oebiotech company using the following protocol. Raw sequencing data were obtained in FASTQ format. Paired-end reads were preprocessed using Trimmomatic software to detect and cut off ambiguous bases (N). It also cut off low-quality sequences with average quality score below 20 using sliding window trimming approach. After trimming, paired-end reads were assembled using FLASH software. Parameters of assembly were: 10 bp of minimal overlapping, 200 bp of maximum overlapping, and 20% of maximum mismatch rate. Sequences were further denoised using QIIME (version 1.8.0) as follows: reads with ambiguous, homologous sequences or below 200 bp were abandoned. Reads with 75% of bases above Q20 were retained. Then, reads with chimera were detected and removed. After processing and generating high-quality sequences, Vsearch software was used to cluster the sequences into operational taxonomic units (OTUs) according to sequence similarity. For 16 S, Greengenes or Silva (Version123) database were used for comparison and annotation of species by RDP Classifier software or Blast software [11–15].

##### ELISA

ELISA measurements of cytokines in blood and intestinal tissue samples were determined by Mouse ELISA Kit (ELISA kits,Multisciences Biotech) according to the manufacturer’s instructions.

#### Western-blot•

100 µg of protein from colon tissue lysates was resolved using SDS-polyacrylamide gel electrophoresis and electro-transferred to polyvinylidene difluoride membranes by transfer. Blots were blocked with 5%BSA for 1 h and then incubated with primary antibodies β-actin (Anti-ACTB rabbit polyclonal antibody, D110001, Sangon Biotech), NF-κB (Recombinant Anti-NF-kB p65 Antibody[E379], ab32536, abcam), p-NF-κB (Anti-NF-kB p65 (phospho S536) Antibody, ab86299, abcam),Dectin-1(Anti-Dectin-1 antibody, ab140039, abcam), TLR4 (Toll-like Receptor 4 (D8L5W) Rabbit mAb (Mouse Specific), 14,358 S, CST), Occludin (Recombinant Anti-Occludin Antibody[EPR20992], ab216327, abcam) and TLR9 (Anti-TLR9 antibody [26C593.2], ab134368, abcam) at 4 °C overnight .Excess antibodies were removed by washing and then Membrane was incubated with antibodies Rabbit Anti-Goat IgG H&L (HRP,Abcam) and Goat Anti-Mouse IgG H&L (HRP polymer,Abcam) for 1 h at room temperature.Blots were detected by ChemiScope mini.

#### Histological examination

To evaluate the severity of histological changes, colon fragments were fixed in 10% formalin (phosphate buffered), paraffin embedded, and histological cuts were stained with hematoxylin and eosin (H&E). The severity of colitis was graded according to four parameters of colonic histological score: (1) the depth of infiltration of inflammatory cells into the mucosal layer; (2) the depth of lesion; (3) the range of lesion; (4) the degree of crypt rupture. Each parameter is scored 0–4 [16].(See in Table 2.)

**Table 2:** The pathological histological scoring

Inflammation	Depth of the lesion	Extent of disease (%)	Crypt destruction	Score
none	none	none	none	0
Mild, inflammatory cells infiltration mucous layer < 1/3	Mucous membrane layer	1–25	1/3 of the basement crypt was destroyed	1
Moderate, inflammatory cell infiltration was more than 1/3 of the full mucosal layer but less than 2/3 of the mucosal layer	Mucosa and submucosa	26–50	2/3 of the basement crypts were destroyed	2
Serious, the infiltration of inflammatory cells exceeded 2/3 of the total mucosal layer	Full-thickness inflammation	51–75	Only part of the epithelium is intact	3
		76–100	All crypts and epithelium are damaged	4

### Statistical analysis

In 16 S rRNA analysis of gut microbiota, alpha diversity of fecal microbiota was assessed by boxplot analysis to determine the differences within microbial community (richness, evenness, phylogenetic diversity), using Wilcoxon/Kruskal Wallis Tests. Based on Bray-Curtis dissimilarity distance matrices, the β-diversity was assessed to reveal the community composition or structure similarity of different samples using Principal coordinate analysis (PCoA) and NMDS analysis, combined with the measurement of Adonis test to estimate statistical differences between groups. Multivariate statistical analysis of microbial species was performed by statistical algorithm (ANOVA/Kruskal Wallis/*t* test/Wilcoxon) to calculate the differential species among different groups and draw the heat map of the differential species. Lefse analysis was used to assess the magnitude of the effect of each species abundance on the differences. Data are expressed as meansSEM. Statistical comparison of two groups was performed using either Student’s t test or Wilcoxon-Mann-Whitney test, and comparison of three groups was analyzed by one-way parametric ANOVA. The statistical analyses were performed using 19.0 SPSS statistical software, and P < 0.05 was considered statistically significant.

## Results

### Effects of TCS exposure on common mice

#### Effects of TCS exposure for 1 week on gut inflammation

**Fig. 1:**
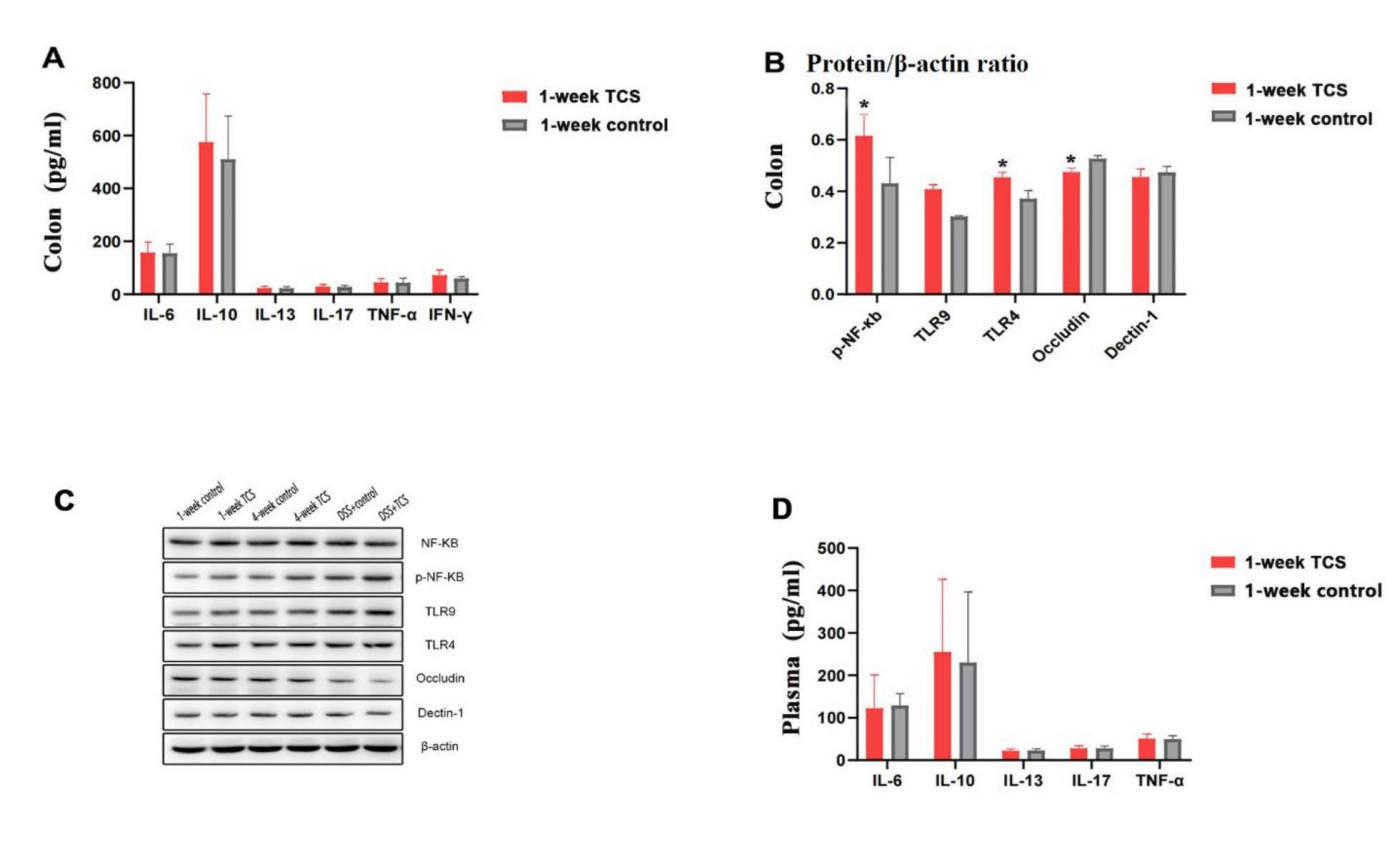
**Contents of factors in mice after 1-week TCS exposure. (A)** The proteinlevels of cytokines in colon tissues examined by ELISA (n = 12/ group). **(B)** the protein levels of cytokines in colon tissues examined by Western blot and were normalized relative to the β-actin (n = 3/group). **(C)**Representative pictures for Western blot runs. According to the sizes of the target bands and comparisons with marker, the target bands were cut out from polyacrylamide.**(D)** Plasma concentration of cytokines examined by ELISA (n = 12/group). The results are expressed as means ± SD. *P < 0.05. The statistical significance of two groups was determined using Student’s *t* test

**Fig. 2:**
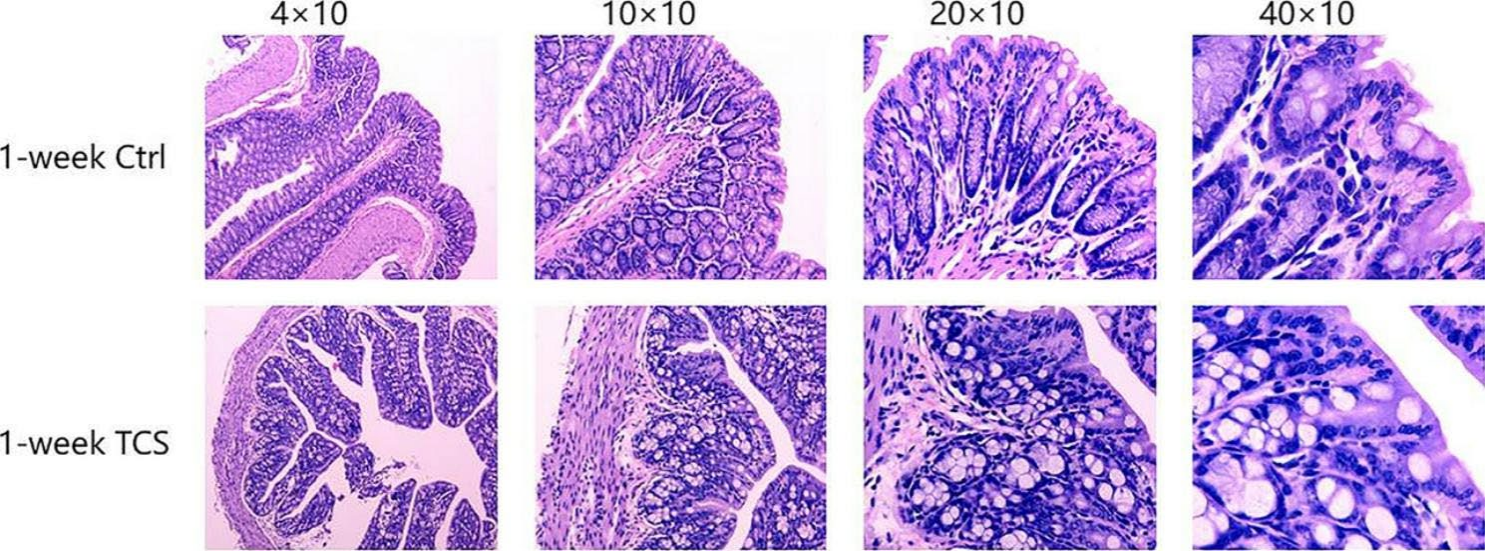
**Colon pathological section of 1-week TCS exposure** HE: magnification, ×40、×100、×200、×400; n = 12/group

Compared with vehicle-treated group, 1-week TCS exposure to mice enhanced the protein levels of p-NF-κb (0.615 ± 0.085 vs. 0.431 ± 0.101) and activation of TLR4 (0.454 ± 0.020 vs. 0.371 ± 0.032), but reduced the levels of Occludin protein in colon (0.475 ± 0.016 vs. 0.527 ± 0.012) (all the *P* < 0.05) (Fig. 1). TCS did not cause intestinal mucosal injury in colon (Fig. 2) and histological scores were 0. Other proinflammatory cytokines in plasma were not elevated (Fig. 1 C) (all the *P* > 0.05) .

#### Effects of TCS exposure for 1 week on gut microbiota in mice

**Fig. 3:**
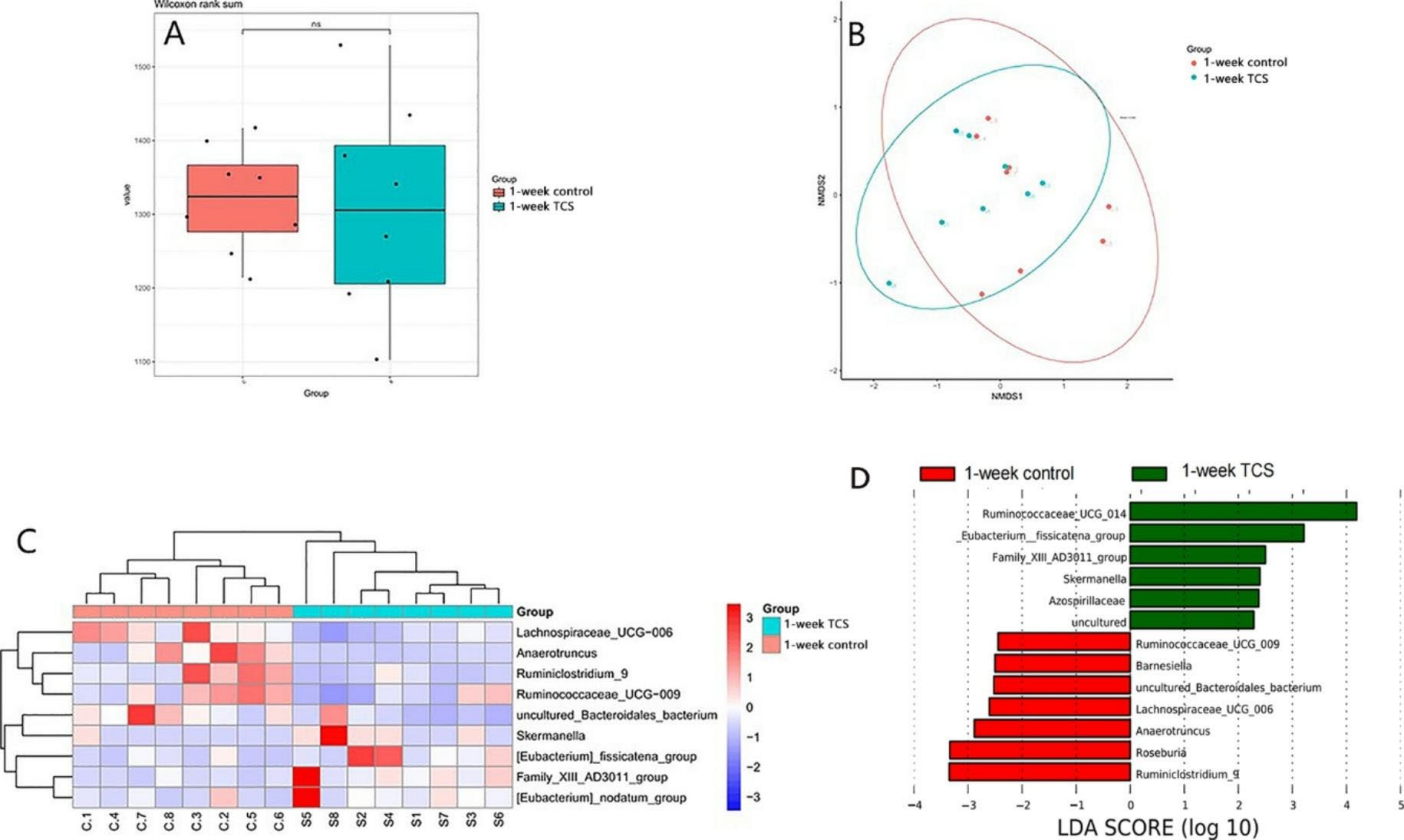
**The effects of TCS exposure for 1 week on gut microbiota in common mice.** Mice were treated with 80 ppm TCS or vehicle alone in diet for 1 week. **(A)** Effect of TCS on α diversity of fecal microbiota, assessed by boxplot. **(B)** Effect of TCS on β diversity of fecal microbiota, assessed by NMDS. **(C)** Heatmap of differences in relative abundance of microbiota at phylum levels. **(D)** Lefse analysis of assessing the magnitude of the effect of each species abundance on the differences. The results are expressed as means ± SD. n = 12/group. ns means no statistical difference. The statistical significance was determined using Wilcoxon-Mann-Whitney test

16 S rRNA sequencing test for stool samples indicated that the α and β diversity of microbiota were not altered by TCS, but the composition of microbiota was. There was no different microbiota at phylum level. At genus level, TCS-treated mice showed higher enrichment of *Eubacterium_fissicatena* and *Eubacterium_nodatum* as well as lower butyrate producing bacteria, *Anaerotruncus*, but less species *Oscillibacter_sp._1–3* in stool samples.(Fig. 3).

.

#### Effects of TCS exposure for 4 weeks on gut inflammation

**Fig. 4:**
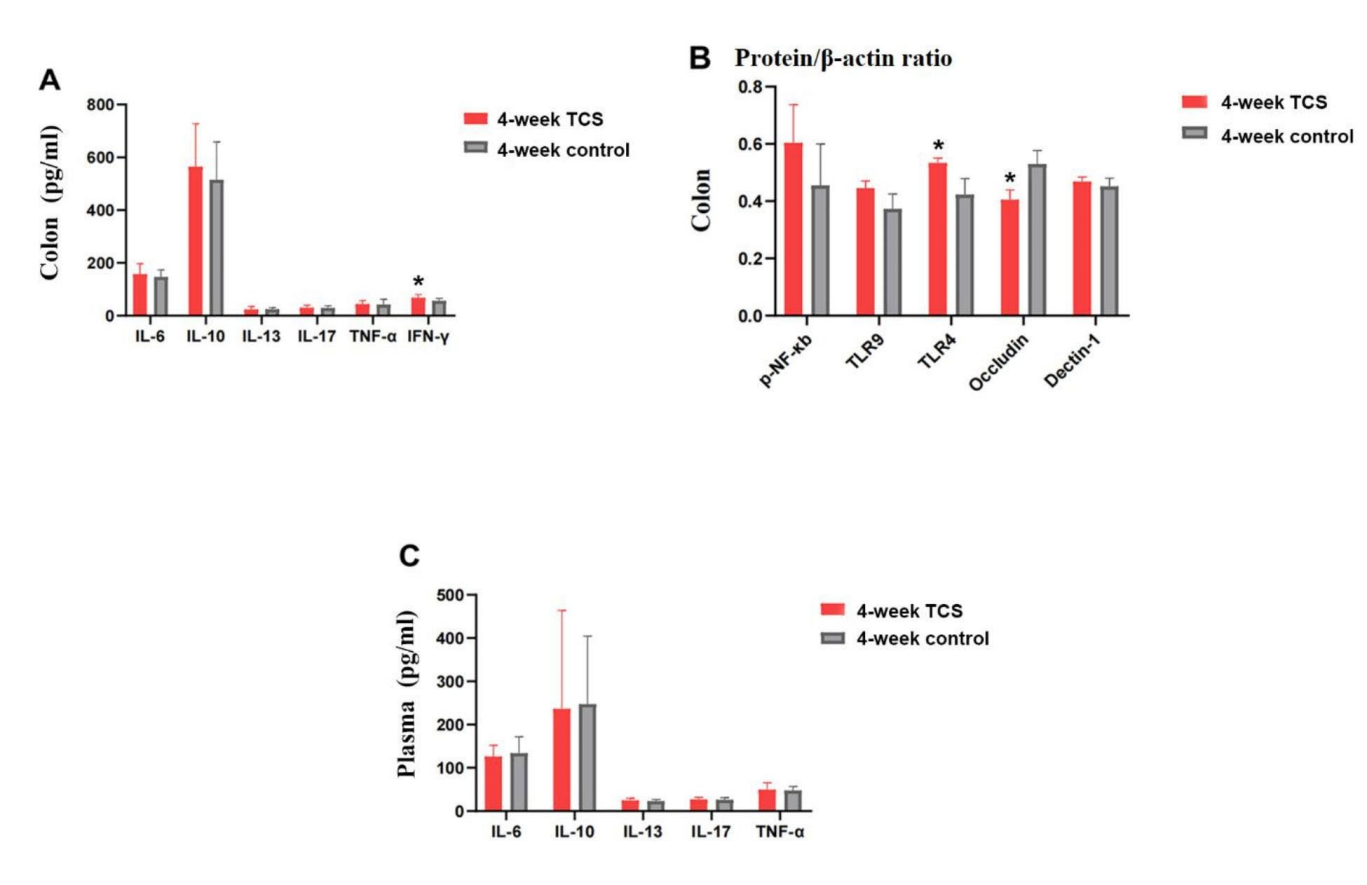
**Contents of factors in mice after 4-week TCS exposure. (A)** The protein levels of cytokines in colon tissues examined by ELISA (n = 12/ group). **(B)** the protein levels of cytokines in colon tissues examined by Western blot and were normalized relative to the β-actin(n = 3/group). **(C)** Plasma concentration of cytokines examined by ELISA (n = 12/group). The results are expressed as means ± SD. *P < 0.05. The statistical significance of two groups was determined using Student’s *t* test

**Fig. 5:**
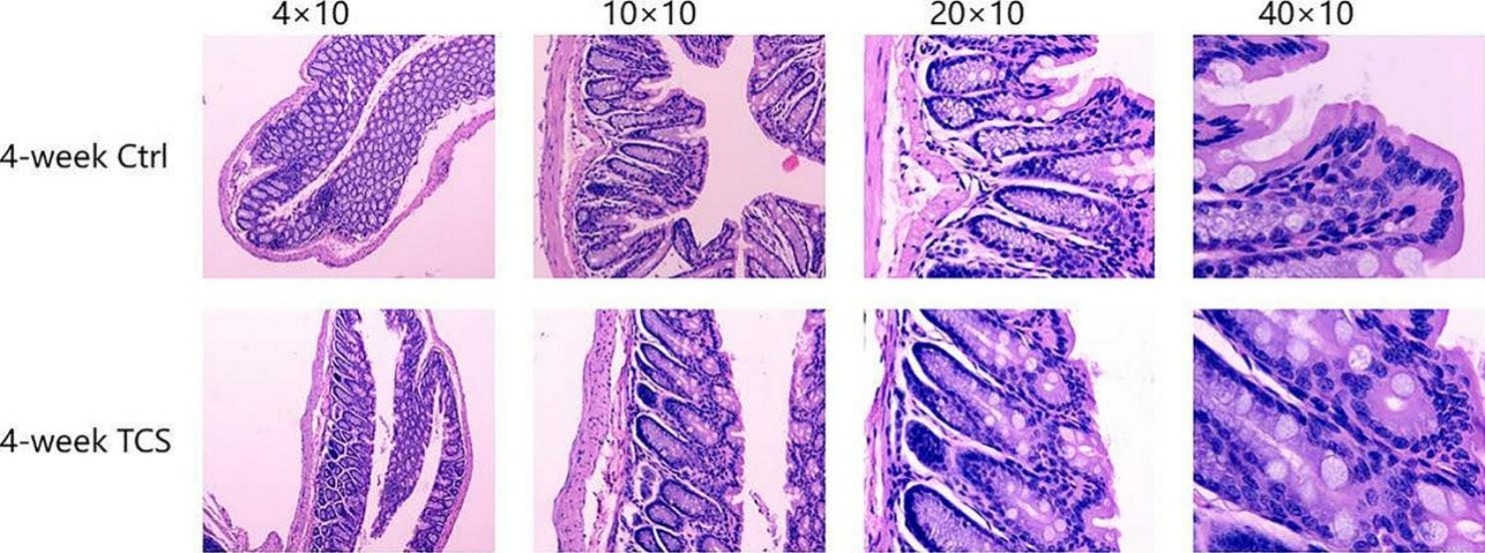
**Pathological section of 4-week TCS exposure** HE: magnification, ×40、×100、×200、×400; n = 12/group

Compared with vehicle-treated group, 4-week TCS-treated mice had lower protein level of occludin (0.405 ± 0.034 vs.0.530 ± 0.047), increased TLR4 (0.534 ± 0.016 vs. 0.423 ± 0.056) and IFN-γ (68.484 ± 11.950 vs. 56.575 ± 9.164) (all the *P* < 0.05) (Fig. 4). TCS exposure for 4 weeks did not cause the mucosa damage in colon and the histological score is 0 (Fig. 5). In comparison to 1-week TCS-treated mice, 4-week TCS-treated mice had less protein level of occludin (0.405 ± 0.034 vs. 0.475 ± 0.016) and more TLR4 (0.534 ± 0.016 vs.0.454 ± 0.020) in colon (both *P* < 0.05).

#### Effects of TCS exposure for 4 weeks on gut microbiota in mice

**Fig. 6:**
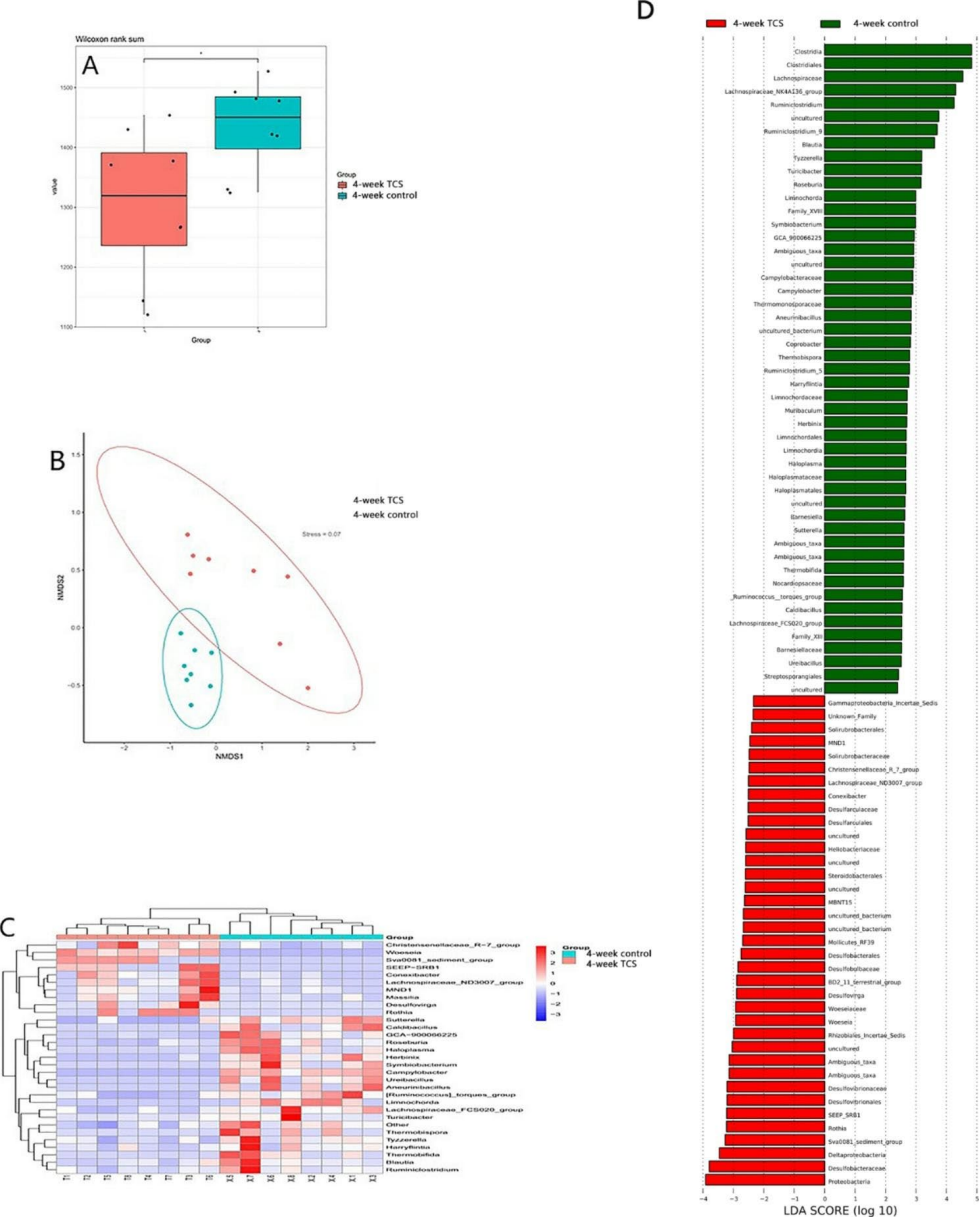
**4-week TCS exposure on gut microbiota.** Mice were treated with 80 ppm TCS in diet for 4 weeks.(A) Effect of TCS on α diversity of fecal microbiota, assessed by boxplot. (B) Effect of TCS on β diversity of fecal microbiota, assessed by NMDS. (C) Heatmap of differences in relative abundance of microbiota at phylum levels. (D) Lefse analysis of assessing the magnitude of the effect of each species abundance on the differences. The results are expressed as means ± SD. n = 12/group. ns means no statistical difference. The statistical significance was determined using Wilcoxon-Mann-Whitney test

The composition of gut microbiota in mice changed significantly after TCS exposure for 4 weeks. The α diversity of microbiota decreased and the β diversity was modulated, assessed by NMDS analysis, showing there was a clear separation. At phylum level, the abundance of *Proteobacteria* augmented; at genus level, Sulfate-reducing bacteria (SRB) increased, including *Sva0081_sediment_group*, *SEEP-SRB1* and *Desulfovirga*, while butyrate-producing probiotic, including, *Roseburia* and *Blautia*, were reduced than the control group. At species level, *Clostridium_sp._ND2* increased, but *Lachnospiraceae_bacterium_28 − 4*, *Lachnospiraceae_bacterium_10-1*and *Lachnospiraceae_bacterium_615* diminished.(all the *P* < 0.05)(Fig. 6).

### Effects of TCS exposure on UC-model mice

#### Effects of TCS exposure on ulcerative colitis in UC-model mice

**Fig. 7:**
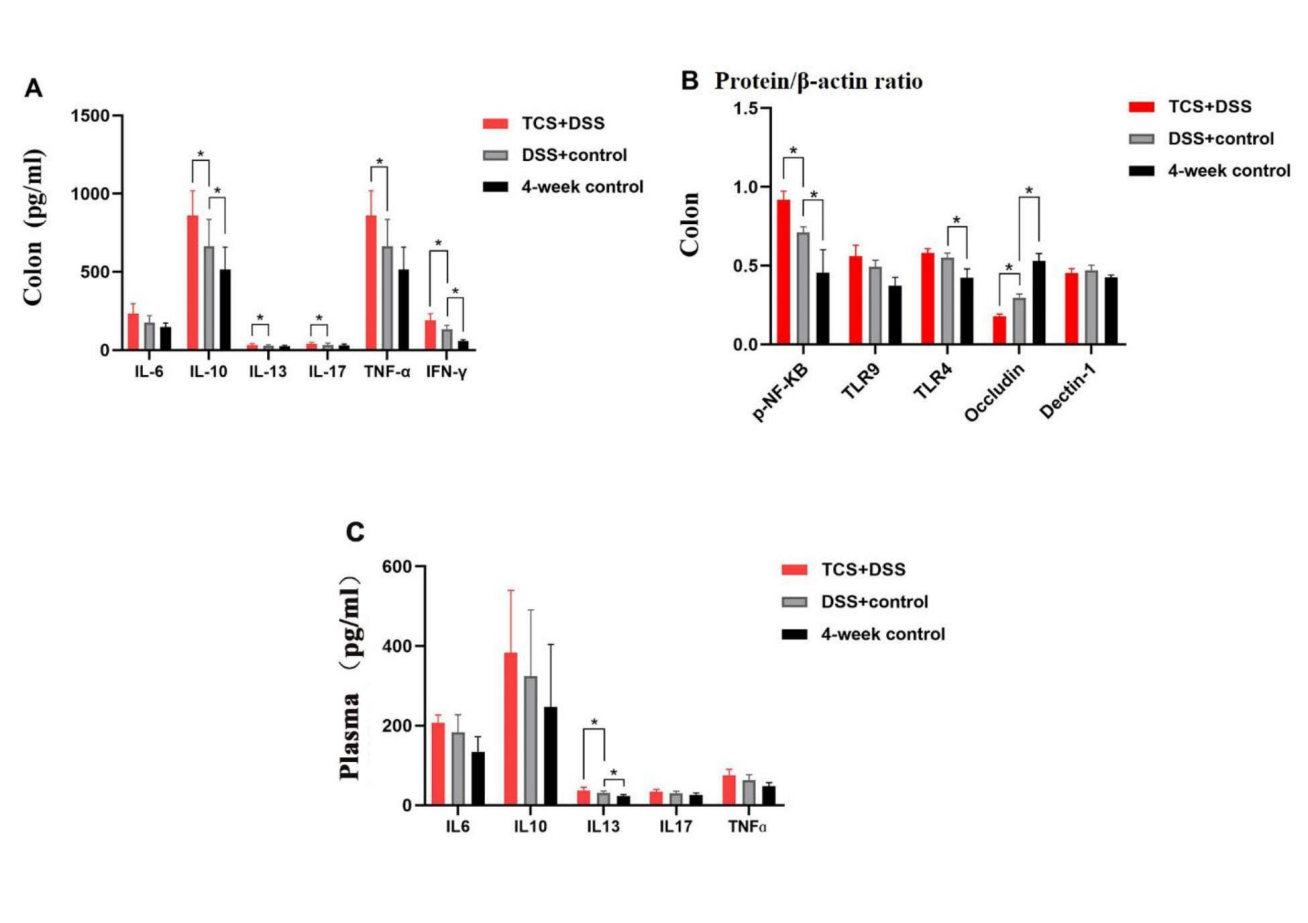
**Contents of factors in DSS-treated mice.** (A) The protein levels of cytokines in colon tissues examined by ELISA (n = 12/ group). (B) the protein levels of cytokines in colon tissues examined by Western blot and were normalized relative to the β-actin (n = 3/group). (C) Plasma concentration of cytokines examined by ELISA (n = 12/group). The results are expressed as means ± SD. *P < 0.05. The statistical significance of two groups was determined using Student’s *t* test

**Fig. 8:**
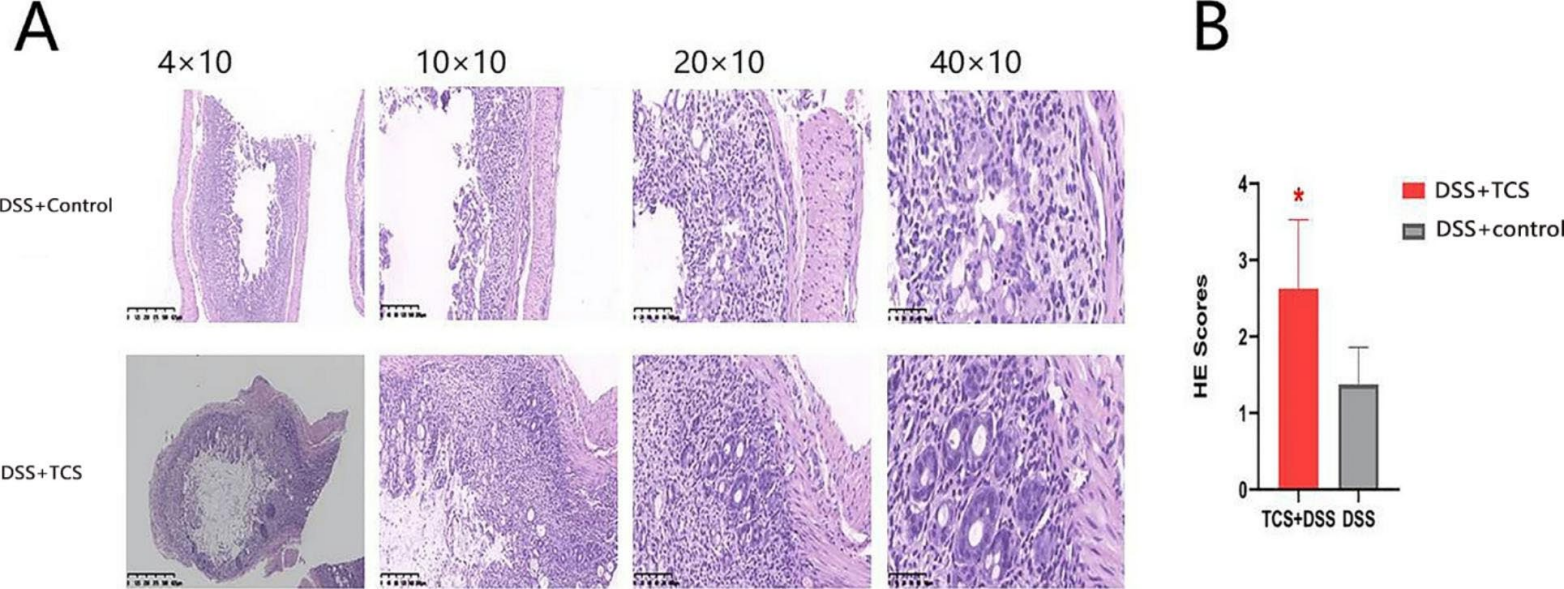
**Colon pathological section and histological scores of DSS-model mice. (A)** HE: magnification, ×40、×100、×200、×400; n = 12/group .**(B)** Histological scores: The results are expressed as means ± SEM. n = 8 per group. *p < 0.05. The statistical significance was determined using Student’s *t* test

Compared with vehicle-treated UC mice, TCS-treated UC mice had higher plasma concentration of IL-13 (but not other cytokines), enhanced the level of IL-10, IL-13, IL-17, TNF-α, IFN-γ and p-NF-κb in colon but reduced the Occludin protein (all the *P* < 0.05) (see Fig. 7). TCS exposure exacerbated the degree of damage to intestinal mucosa and crypt, infiltration of inflammatory cells and atypia of glandular cells. Low grade intraepithelial neoplasia appeared in colon in TCS-treated UC mice. The histological injury score was higher than that in vehicle-treated UC group. (2.625 ± 0.904 vs. 1.375 ± 0.484, *P* < 0.05, see Fig. 8)

#### Effects of TCS exposure on gut microbiota in UC-model mice

**Fig. 9:**
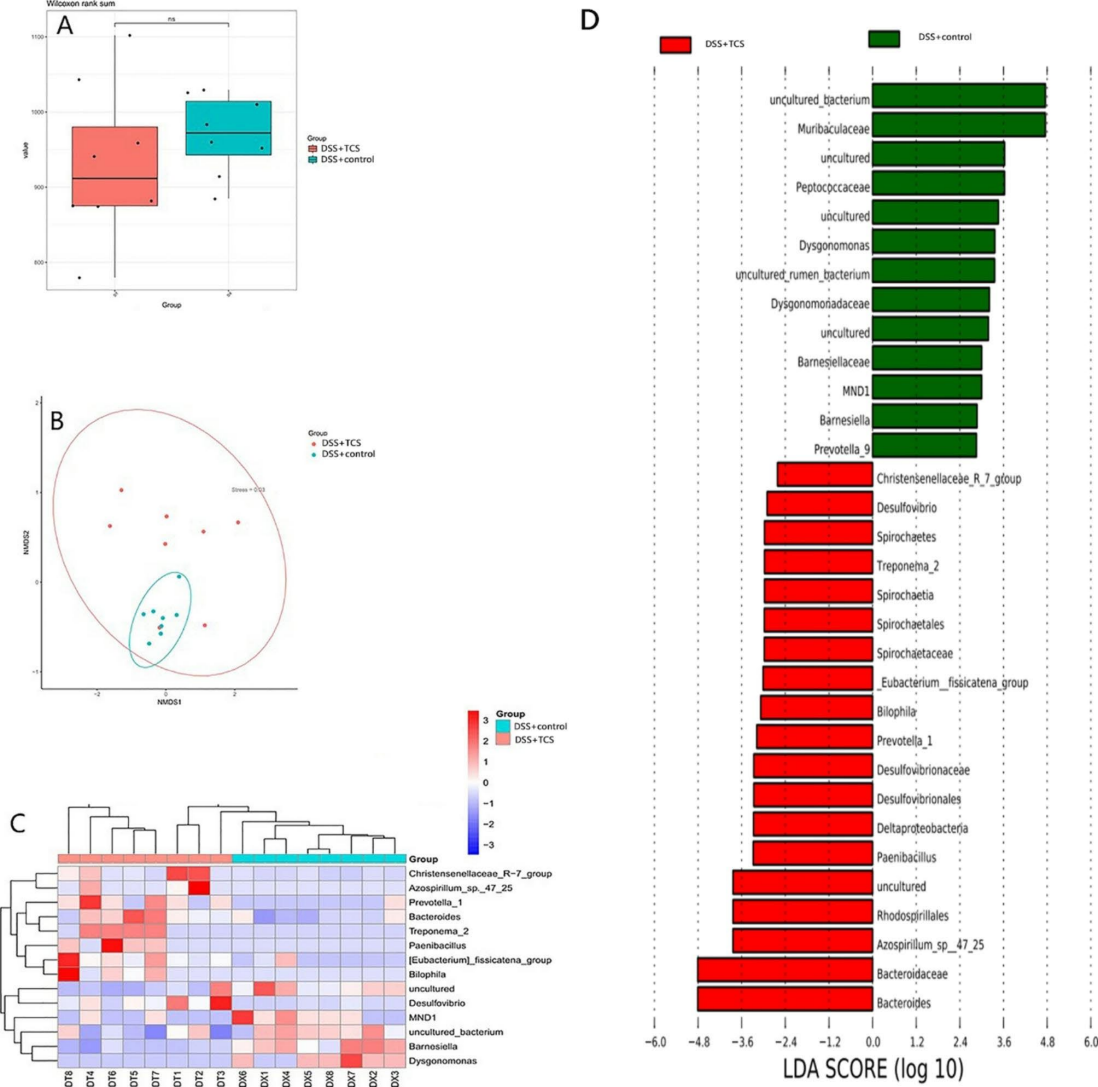
**TCS exposure on gut microbiota in DSS-model UC mice.** Mice were treated with 80 ppm TCS in diet for 3 weeks. At the beginning of the 4th week, all the mice were treated with 2% DSS in drinking water for 1 week to induce UC, during which the TCS treatment remained the same. **(A)** Effect of TCS on α diversity of fecal microbiota, assessed by boxplot. **(B)** Effect of TCS on β diversity of fecal microbiota, assessed by NMDS. **(C)** Heatmap of differences in relative abundance of microbiota at phylum levels. **(D)** Lefse analysis of assessing the magnitude of the effect of each species abundance on the differences. The results are expressed as means ± SD. n = 12/group. ns means no statistical difference. The statistical significance was determined using Wilcoxon-Mann-Whitney test

In UC-model mice, the composition of gut microbiota was altered by TCS exposure but there was no difference in the α and β diversity. At phylum level, TCS exposure increased *Acidobacteria* and *Spirochaetes*. At genus level, TCS-treated UC mice had more abundance of SRB (*Desulfovibrio*), *Bilophila*, *Prevotella_1* and *Bacteroidesas*, and less *Barnesiella*. Species *Bacteroides_gallinaceum* and *Bacteroides_acidifaciens* increased while *Bifidobacterium_animalis* and *uncultured_Alistipes_sp.* decreased than vehicle-treated UC mice (all the *P* < 0.05) (see Fig. 9).

## Discussion

In this study, the diversity and composition of gut microbiota were changed by TCS exposure both in healthy and UC-model mice to some extent. The characteristics of microbiome alteration were that TCS reduced the abundance of butyrate-producing probiotics (e.g.*Lachnospiraceae* [17], *Anaerotruncus*, *Roseburia* and *Alistipes*, etc.), but increased detrimental bacteria, including the SRB (e.g. *Sva0081_sediment_group, SEEP-SRB1, Desulfovirga*, etc.) and *Bacteroides*. As the main energy provider for intestinal epithelial cells, butyrate could inhibit the signaling pathway of pro-inflammatory cytokines, and augment the intestinal barrier function by enhancing tight-junctions [18]. Previous studies showed that in UC patients, the level of butyrate-producing bateria and butyrate concentration went down in gut [19]. Microbiota-derived butyrate is critical to maintaining intestinal homeostasis, and dysbiosis of the microbiota in disease states commonly diminishes butyrate levels through decreasing butyrate-producing bacteria, notably in inflammatory bowel diseases (IBD) [20]. And supplement of butyrate-producing bacteria as probiotics showed therapeutic benefits in several diseases [21]. *Bifidobacterium_animalis* could alleviate UC and increase intestinal cell survival [22]. *Alistipes* had protective effects against colitis [23]. The reduction of butyrate-producing bacteria in our results suggest that TCS exposure could potentially impact DSS severity by attenuating the protective effect of probiotics on gut health. However, *Bacteroides* were proved to develop colitis via promotion of IgA and IgA + B cells production, representing extensive systemic secondary immunology response in the lesions [24, 25]. Generally, SRB are normal gut microbiota associated with the hydrogen sulfide (H_2_S) production, but their augment and translocation could activate immune response in the gut and accelerate colitis development by upregulating the Th17 and Treg profiles of cytokine production. Besides, over SRB generated higher levels of H_2_S which jeopardized the intestinal epithelium cells directly [26]. It is believed that the dysregulation of microbiota in UC patients and its interaction with the host were crucial mechanisms resulting in immune response and inflammatory damage [27]. In this study, the inflammatory responses in UC-model mice were exaggerated by TCS exposure with higher histological injury score and significantly increased Th1-associated cytokines (TNF-α, IFN-γ ), Th2 cytokines (IL-10, IL-13) and Th17 cytokines (IL-17) in colon.

Our results are in agreement with previous study conducted by Yang et al. showing that gut microbiota contributes to the proinflammatory effects of TCS [4]. They showed that the exposure of TCS (80 ppm in diet) caused adverse effects on colonic inflammation and colon cancer, which was associated with reduced diversity of the gut microbiota and decreased abundance of beneficial gut bacteria such as *Bifidobacterium* as well as TLR4 signaling. They also found that the enhancing effect of TCS on basal inflammation was abolished in germ-free and Tlr4−/− mice, which confirms that gut microbiota and TLR4 were required for the biological effect of TCS. Similarly, our study showed that TCS exposure reduced the protein levels of Occludin and increased TLR4, p-NF-κb and IFN-γin gut tissues. Commonly, TLR4 is expressed at the lateral surface of intestinal epithelial cells as innate immune receptor and strictly regulated in colon, which is physically separated from the microbiome [28]. Gut microbiota would enter the intestinal epithelial across injured mucosal barrier to activate TLR4-related immune response when intestinal mucosal barrier was damaged and the permeability increased [29]. Overexpression of TLR4 could activate NF-κb signaling pathway and induce a series of proinflammatory cytokines [30]. Occludin, as an intestinal mucosal tight junction protein, is responsible for dysfunction of intestinal barrier and microbial penetration [31]. But there are some difference between the two studies. In the previous study, mice were treated with TCS for 3 weeks and the inflammatory injury in gut was observed showing that higher plasma concentration of IL-6 and enhanced gene expression of Il-6 in colon as well as exaggerated crypt damage in colon. However, our study suggested that the outset that the effects of TCS on gut might be earlier. We set up 1-week and 4-week TCS treatment groups. TCS treatment for 1 week caused higher protein levels of p-NF -κ B and TLR4 in gut, compared with which, 4-week TCS treatment increased the protein level of TLR4. On the other hand, unlike the previous study, during either 1 week or 4 weeks, TCS treatment did not increase the plasma concentration and the protein level in gut of IL-6, and no mucosal damage was observed in the HE-stained histological sections of gut tissue. The intestinal damage caused by TCS appeared to develop more slowly in normal mice. In addition, our results complemented the effect of TCS in promoting tumorigenesis. TCS-treated UC-model mice showed atypia of glandular cells towards low grade intraepithelial neoplasia but mice without TCS treatment did not, while in Yang’s azoxymethane (AOM)/DSS-induced colon cancer model in mice, TCS treatment (80 ppm in diet) enhanced overall mortality, average tumor number and tumor size.

There was limitation that the metabolism of TCS in mice might be not wholly equivalent to it in human beings, but our study was an approximate method as possible. Our dose regimen was based on the detected average human exposure levels of TCS [9]. Beyond that, opposing views held that within added concentrations of TCS set by officially prescribed, concerns about the safety of products containing TCS were unnecessary. Whereas, we contend the amount of TCS we exposed to is much more than that in our daily products. TCS has been listed in the top ten river pollutants in the world. In addition to toothpastes and hand sanitizer, it also was found in surface soils, drinking water, plants and animals, which could be transferred to humans through the biological food chain [32]. Furthermore, the pro-colitis effects of TCS in this study should be of concern. Especially for patients with UC, who could be more susceptible to TCS, we suggest they would better select products without TCS. TCS exposure matters the occurrence and development of colitis. It is urgent to conduct more studies on dose- and time- dependent effects of TCS on human gut health.

## Conclusion

Triclosan exposure induced disturbance of gut microbiota and exaggerated experimental colitis in mice. And changes of composition of gut microbiota were characterized as the increase of harmful bacteria, including sulfate-reducing bacteria and *Bacteroides*, and the reduction of protective probiotics, butyrate-producing bacteria.

## Electronic supplementary material

Below is the link to the electronic supplementary material.

Supplementary Material 1

## Data Availability

the data related to this study has been uploaded to the NCBI database. **BioProject ID**: PRJNA801918. The link is https://www.ncbi.nlm.nih.gov/bioproject/PRJNA801918.

## Abbreviations

DSS: Dextransulfatesodium
TLR4: Toll-like receptor 4
p-NF-κb: Nuclear factor κb
UC: Ulcerative colitis
TCS: Triclosan
H&E: hematoxylin and eosin
SRB: Sulfate-reducing bacteria
H_2_S: hydrogen sulfide
TJ: Tight junction
