# Supplementary material for: Triclosan exposure induced disturbance of gut microbiota and exaggerated experimental colitis in mice
Source: BMC Gastroenterol. 2022 Nov 18;22:469. doi: 10.1186/s12876-022-02527-z (PMC9675201; doi:10.1186/s12876-022-02527-z)
Supplement: Supplementary file 1 — Supplementary Material 1 [file 12876_2022_2527_MOESM1_ESM.docx]

| Groups  Factors | 1-week control | 1-week TCS  **Western blot bands** | 4-week control | 4-week TCS | DSS+ control | DSS+TCS | 4-week TCS | DSS+ control | DSS+  TCS |
| --- | --- | --- | --- | --- | --- | --- | --- | --- | --- |
| NF-κb | 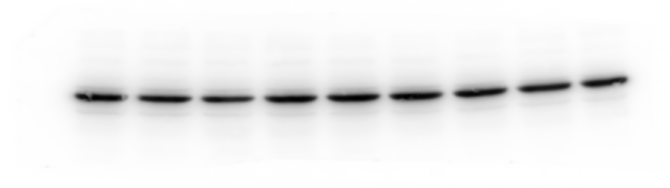1. | | | | | | | | |
|  | 2.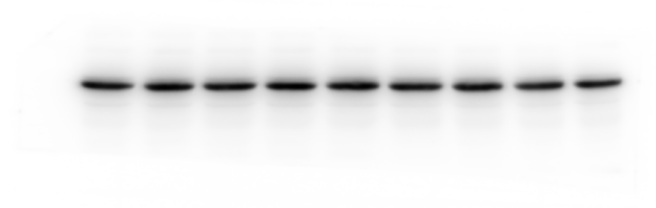 | | | | | | | | |
| P-NF-κb | 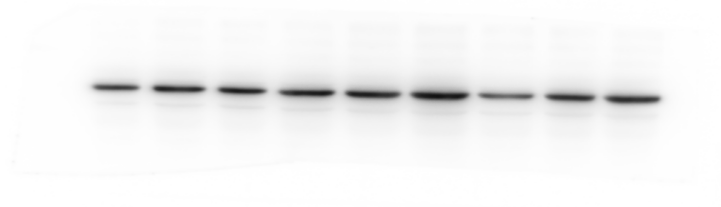1. | | | | | | | | |
|  | 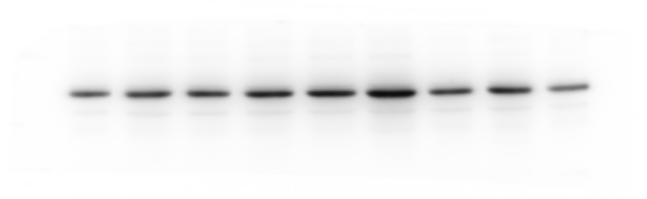2. | | | | | | | | |
| TLR9 | 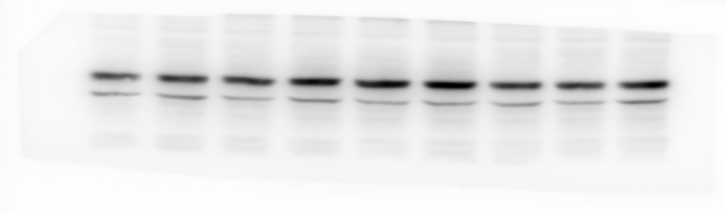1. | | | | | | | | |
|  | 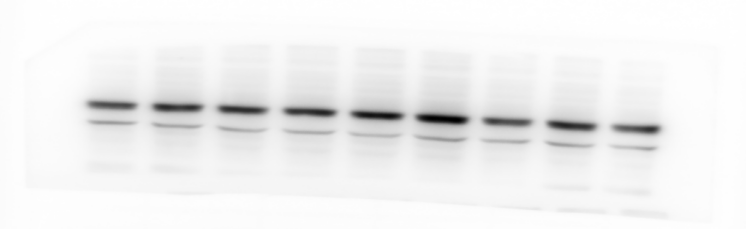2. | | | | | | | | |
| TLR4 | 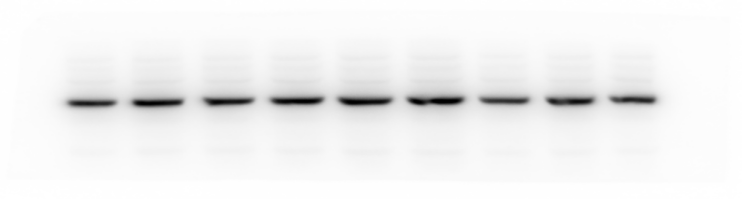1. | | | | | | | | |
|  | 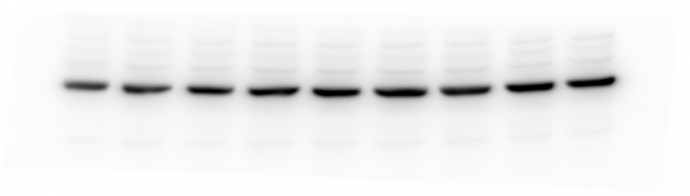2. | | | | | | | | |
| Occludin | 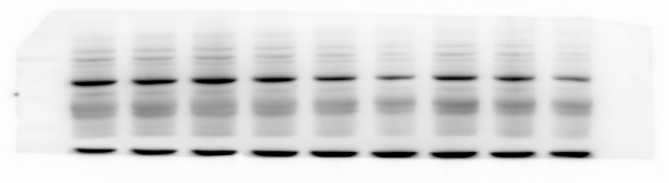1. | | | | | | | | |
|  | 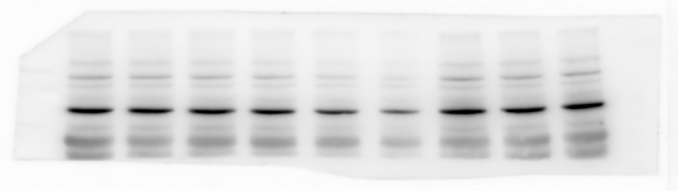2. | | | | | | | | |
| Dectin-1 | 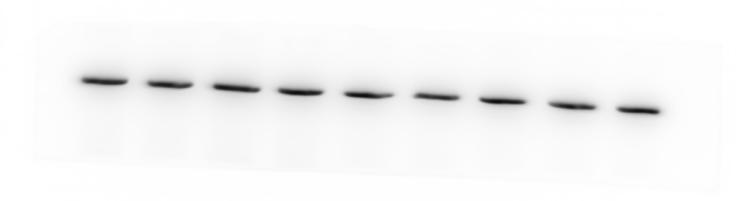1. | | | | | | | | |
|  | 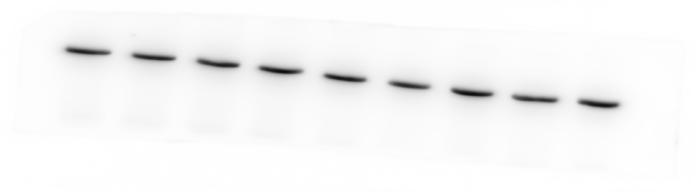2. | | | | | | | | |
| β-actin | 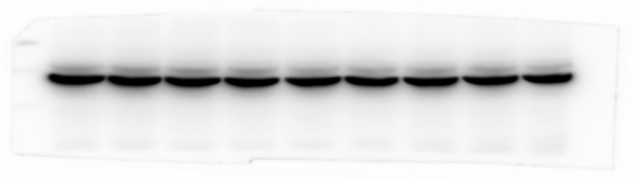1. | | | | | | | | |
|  | 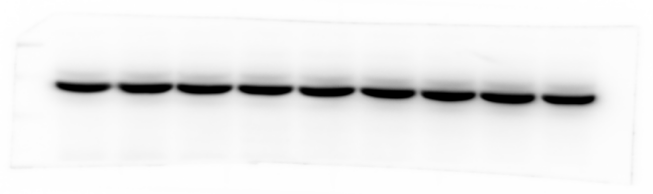2. | | | | | | | | |
